# Supplementary material for: The Impact of Soil pH on Heavy Metals Uptake and Photosynthesis Efficiency in Melissa officinalis, Taraxacum officinalis, Ocimum basilicum
Source: Molecules. 2022 Jul 22;27(15):4671. doi: 10.3390/molecules27154671 (PMC9331646; doi:10.3390/molecules27154671)
Supplement: Supplementary file 1 [file molecules-27-04671-s001.zip › molecules-1752557-supplementary.pdf]

The impact of soil pH on heavy metals uptake and photosynthesis efficiency in Melissa Officinalis, Taraxacum officinalis, Ocimum basilicum

Dorota Adamczyk-Szabela[1]* and Wojciech M. Wolf[2]

[1] Lodz University of Technology, Institute of General and Ecological Chemistry, 90-924 Lodz, Zeromskiego 116, Poland; dorota.adamczyk@p.lodz.pl (D.A-S.);
[2] Lodz University of Technology, Institute of General and Ecological Chemistry, 90-924 Lodz, Zeromskiego 116, Poland; wojciech.wolf@p.lodz.pl (W.M.W.);
* Correspondence: dorota.adamczyk@p.lodz.pl

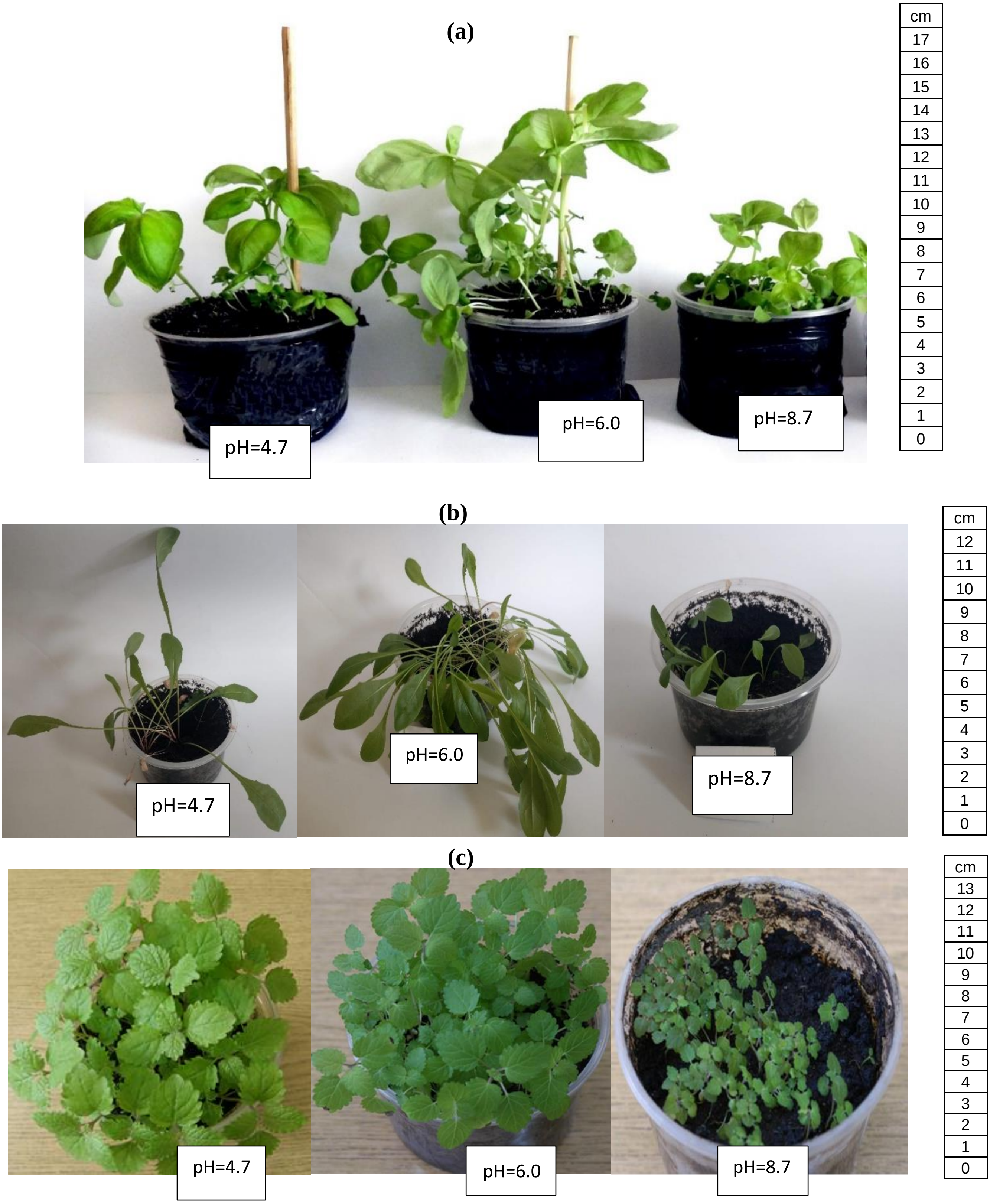

Figure S1. Basil (a), dandelion (b) and lemon balm (c) plants are grown in different soil pH.

Table S1. Metals content in certified reference materials (p = 0.95, n = 5).

Metal	Certified value µg/g	Found µg/g	Recovery %
**Manganese**	191 ± 12	180 ± 10	94
**Copper**	7.77 ± 0.53	7.08 ± 0.41	91
**Zinc**	33.5 ± 2.1	34.2 ± 1.1	102
